# Optimising conservative management of chronic low back pain: study protocol for a randomised controlled trial

**DOI:** 10.1186/s13063-017-1913-8

**Published:** 2017-04-20

**Authors:** Katherine J. Simson, Clint T. Miller, Jon Ford, Andrew Hahne, Luana Main, Timo Rantalainen, Wei-Peng Teo, Megan Teychenne, David Connell, Guy Trudel, Guoyan Zheng, Gary Thickbroom, Daniel L. Belavy

**Affiliations:** 1 Institute for Physical Activity and Nutrition, School of Exercise and Nutrition Sciences, Deakin University, Geelong, VIC 3220 Australia; 2 Advance HealthCare, 157 Scoresby Rd, Boronia, VIC 3155 Australia; 3 La Trobe University, Kinsbury Drive, Bundoora, VIC 3083 Australia; 4 Imaging@Olympic Park, AAMI Park, 60 Olympic Boulevard, Melbourne, VIC 3004 Australia; 5 University of Ottawa, 505 Smyth Road, Ottawa, ON K1H 8M2 Canada; 6 University of Bern, Stauffacherstrasse 78, Bern, Bern CH-3014 Switzerland; 7 Burke Medical Research Institute, Weill-Cornell Medical College, New York, NY USA

**Keywords:** Low back pain, Exercise therapy, Motor control

## Abstract

**Background:**

Lower back pain is a global health issue affecting approximately 80% of people at some stage in their life. The current literature suggests that any exercise is beneficial for reducing back pain. However, as pain is a subjective evaluation and physical deficits are evident in low back pain, using it as the sole outcome measure to evaluate superiority of an exercise protocol for low back pain treatment is insufficient. The overarching goal of the current clinical trial is to implement two common, conservative intervention approaches and examine their impact on deficits in chronic low back pain.

**Methods/design:**

Forty participants, 25–45 years old with chronic (>3 months), non-specific low back pain will be recruited. Participants will be randomised to receive either motor control and manual therapy (*n* = 20) or general strength and conditioning (*n* = 20) exercise treatments for 6 months. The motor control/manual therapy group will receive twelve 30-min sessions, ten in the first 3 months (one or two per week) and two in the last 3 months. The general exercise group will attend two 1-hour sessions weekly for 3 months, and one or two a week for the following 3 months. Primary outcome measures are average lumbar spine intervertebral disc T2 relaxation time and changes in thickness of the transversus abdominis muscle on a leg lift using magnetic resonance imaging (MRI). Secondary outcomes include muscle size and fat content, vertebral body fat content, intervertebral disc morphology and water diffusion measured by MRI, body composition using dual energy X-ray absorptiometry, physical function through functional tests, changes in corticospinal excitability and cortical motor representation of the spinal muscles using transcranial magnetic stimulation and self-reported measure of pain symptoms, health and disability. Outcome measures will be conducted at baseline, at the 3-month follow-up and at 6 months at the end of intervention. Pain, depressive symptomology and emotions will be captured fortnightly by questionnaires.

**Discussion:**

Chronic low back pain is ranked the highest disabling disorder in Australia. The findings of this study will inform clinical practice guidelines to assist with decision-making approaches where outcomes beyond pain are sought for adults with chronic low back pain.

**Trial registration:**

Australian New Zealand Clinical Trials Registry, ACTRN12615001270505. Registered on 20 November 2015.

**Electronic supplementary material:**

The online version of this article (doi:10.1186/s13063-017-1913-8) contains supplementary material, which is available to authorized users.

## Background

Low back pain is a global health issue affecting up to 84% of adults [1]. In Australia, it bears the greatest cost to society in terms of disability and lost productivity compared to any other disease [2]. Chronic non-specific lower back pain represents approximately 23% of reported low back pain; however, it consumes a large fraction of the $9.17 billion Australia spends annually on back pain [3, 4].

Meta-analysis has shown that whilst exercise in general is beneficial for reducing back pain [5], there is no clear evidence for superiority of one exercise protocol above another. As pain has been identified as multifactorial, a subjective method of measuring pain with ‘self-reported’ tools as the sole outcome to evaluate the superiority of a particular exercise protocol for chronic low back pain is insufficient. Other decrements can be measured to detect the effectiveness of a particular exercise protocol that would provide further information for the selection of treatment approaches for chronic low back pain. The overarching goal of the current randomised clinical trial is to implement two common, but distinct, conservative intervention approaches and examine their impact on a variety of deficits in chronic low back pain. This information will then contribute to guidelines for advising clinicians on expected physical, functional and psychological outcomes following each of these typical interventions for chronic low back pain.

Changes to the intervertebral discs (IVDs) of the lumbar spine are considered to be a common trigger for generating pain [6, 7], and back pain is associated with IVD degeneration [8–10]. Whilst we have an in-depth understanding of what loading protocols and activities might damage the IVD [11, 12], it is not clear whether habitual activities or exercise regimens lead to positive IVD adaptations. In a recent review of the literature, it was proposed that specific exercise and loading protocols might lead to an anabolic response in the IVDs [12]. Whilst there is research in animals to show that IVD anabolism can occur in response to ambulatory exercise [13], currently there is no compelling evidence that the IVDs can respond positively to exercise in humans. Recently, long-distance runners were found to have better IVD characteristics than non-athletes; however, this was tested only through a cross-sectional study [14]. A randomised controlled trial of exercise targeting the IVD is necessary to provide evidence as to whether the IVD can respond positively to exercise and what types of exercise might be optimal. Beyond feeding into guidelines for the prevention and management of IVD injury and spinal pain, this trial will address underlying questions of whether the IVD, similar to muscle [15] and bone [16], can positively respond to exercise. A primary aim of this study is to conduct a pilot study to examine whether a specially designed exercise protocol can result in positive adaptations in the lumbar IVDs.

The time required for a measurable response of the IVD to loading in humans is not clear. Given data indicating that adaptation of tendon tissue in humans requires a month or more [17], and that bone typically requires 6 months to 1 year before a measurable change in bone density in response to exercise can be detected [18, 19], we decided to implement a 6-month intervention period with follow-up at 3 and 6 months to track the time course of adaptation.

Beyond this, in chronic low back pain there is evidence of impaired motor functioning of specific trunk muscles. It has been previously acknowledged that the deep muscles of the trunk, in particular the transversus abdominis, provide an anatomical and biomechanical stiffness of the lumbar spine, potentially providing a protective effect to the spine [20–23]. The function of the transversus abdominis has been found to be negatively impacted in patients with low back pain. There is impaired ability to contract this muscle in athletic populations with back pain [24, 25] and also delay of its activation in postural adjustments [26]. This is thought to reduce stability within vertebral segments and predispose to future injury [25, 27]. There is little information in the literature, however, as to whether performing a targeted rehabilitation protocol restores the function of these deep muscles. Despite the number of cross-sectional studies suggesting a role of the transversus abdominis in back pain, we have identified only one randomised trial in which the effectiveness of specific muscle activation exercises on the function of the transversus abdominis was measured [28], and this study did not find evidence for efficacy of this training program for improving transversus abdominis muscle activation timing. There is evidently a paucity of data on whether the purported targets of specific motor control training are indeed achieved [29]. The second primary aim of this trial is to examine the impact of a specific motor control program on activation of the transversus abdominis in chronic low back pain.

Furthermore, a number of other deficits have been commonly identified in low back pain. Spinal muscle atrophy, strength loss [30–34], reduced activity levels [32, 35–37], reduced cardiovascular fitness [38, 39], muscular endurance and flexibility [40, 41] as well as decreased corticospinal excitability of the paravertebral muscles as measured by transcranial magnetic stimulation [42], poorer sleep quality [43], depressive symptoms [44] and fear of movement [45] have all been demonstrated in patients with low back pain and are thought to contribute to reductions in daily activity and chronicity of pain. With so many indicators of chronic lower back pain being identified, there is a scarcity of data in the literature as to whether various exercise protocols are more beneficial for improving these outcomes. For example, multifidus muscle size has shown both increases and decreases with exercise interventions [46–48], making it difficult to draw conclusions to inform clinical practice. The secondary aim of this study is to examine the impact of the exercise protocols on additional deficits of low back pain previously not measured, including muscle atrophy, physical function parameters, central nervous system adaptations and psychological factors in chronic low back pain.

## Methods/design

### Design

**Fig. 1:**
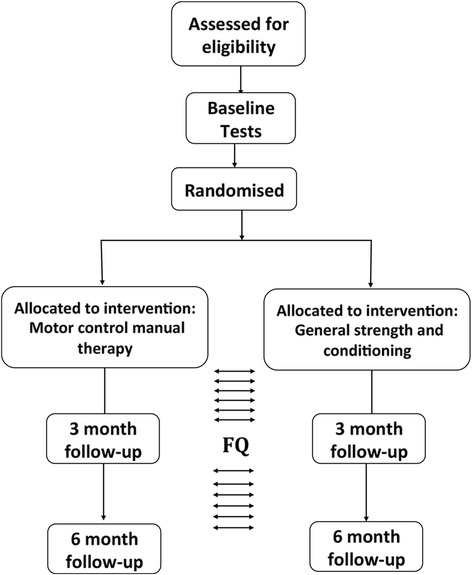
Trial flowchart outlining participant testing and intervention duration. Fortnightly questionnaires (indicated by *FQ*; depressive symptoms, pain, Positive and Negative Affect Schedule (*PANAS*)) are emailed throughout study. At baseline, 3-month and 6-month follow-up the following tests are performed: anthropometry, dual energy X-ray absorptiometry, VO_2_ peak, one-repetition maximum (*1RM*) muscular strength, 70% 1RM local muscular endurance (*LME*), Trunk LME, transcranial magnetic stimulation, magnetic resonance imaging, and questionnaires are given (Sports Injury Rehabilitation Beliefs Survey, International Physical Activity Questionnaire, medication, Oswestry Disability, Medical Outcome Trust Short Form-36 Health Survey V1, Pittsburgh Sleep, Tampa Kinesiophobia, Endicott Work Productivity, PANAS, depressive symptoms and pain)

The randomised clinical trial is summarised in Fig. 1. The study was approved by the Deakin University Human Research Ethics Committee on 12 October 2015 and is registered with the Australian New Zealand Clinical Trials Registry (ACTRN12615001270505). Reporting of the study will adhere to the Consolidated Standards of Reporting Trials (CONSORT) guidelines [49]. Participants will be randomised into either a motor control and manual therapy treatment group or a general strength and conditioning group. The SPIRIT checklist for this study protocol can be accessed as Additional file 1.

### Participants

Volunteers aged 25–45 years [50] with non-specific chronic (>3 months) lower back pain between the T12 vertebra and the gluteal fold with a pain intensity from 2 to 8 (inclusive) on a numerical rating scale (NRS) of 0 to 10 will be eligible to participate in the study. The age range was restricted, as the response of IVD cells to loading depends [50] on whether these cells come from younger versus older or degenerated versus non-degenerated IVDs. The data [51–53] in the literature indicate that the IVD is still maturing in the third decade of life. Beyond approximately the age of 35, there is a normal age-related decline in IVD tissue, and IVD degeneration becomes more common [53]. Volunteers will be excluded from participation if they report a history of spinal surgery, history of traumatic injury to the spine (e.g. fractures, car accident), symptoms of cauda equina syndrome, planning or possibility of invasive treatment (surgery, injections) in the next 6 months, known structural scoliosis, symptoms of nerve root compression including radicular pain or pins/needles/numbness in a dermatomal distribution or non-musculoskeletal causes of back pain (as determined through magnetic resonance imaging (MRI) at baseline). They will also be excluded if they are unable to communicate easily in English, have current parallel treatment for back pain, report more than 150 min/week of moderate-vigorous exercise, participate in formal organised sport, participate in gym-based exercise more than one day per week, have a compensable claim for their back pain, are pregnant, possibly pregnant or considering pregnancy in the next 6 months, have given birth in the last 9 months, are currently breast-feeding, are a current smoker, have known anaemia, a body mass of more than 120 kg or a history (including familial history) of seizures or epilepsy, stroke, head injury or brain-related disorders, are on medication for mental illness, have metal implants or electronic implants unsuitable for MRI or dual energy X-ray absorptiometry (DXA), have had nuclear medicine (radioactive contrast agent) performed in the last 3 months or are unable to attend two 1-hour training sessions per week over a 6-month period and three 3-hour testing days plus an additional MRI scan.

### Recruitment

Participants will be recruited from the general public between inner Melbourne and the eastern suburbs. Print and web-based advertisements will be distributed to local businesses and medical/health centres, within Deakin University staff and students via email and posted on social media. A study website will be provided, and interested volunteers will be required to register through this website. Study team members will then make contact via telephone where applicants will be screened against inclusion and exclusion criteria and made aware of the study time frame and commitment. If the applicant passes telephone screening, he/she will be sent the study plain language statement consent form, explaining study requirements, procedures and time commitments, and will be required to visit a general practitioner (GP) to gain clearance to participate.

### Sample size

Prior data on the impact of interventional exercise on the intervertebral disc do not exist. Recent cross-sectional work on habitual (minimum 5-year history) runners and sedentary individuals showed a 9–11% difference in lumbar IVD T2-time between these collectives [14]. For this first interventional study on exercise and IVD adaptation, we targeted a 0.5% change in lumbar IVD T2-time as a minimum detectable effect size. This compares to a 0.2–0.3% loss of lumbar IVD T2-time per 1 year with normal aging above the age of 30 [54]. Data from the research group (Belavy et al., unpublished observations) show a mean (SD) lumbar IVD T2-time of 100.6 (12.4) with a correlation of 0.982 (coefficient of variation (CV): 1.7%) between repeated measures. To detect a 0.5% difference, or an effect size of 0.0406, on a group*time interaction in the change in lumbar IVD T2-time with two study arms and three measurement time-points, and assuming an alpha of 0.05 and a power of 0.8, a sample size of 36 would be required. Assuming 10% dropouts, this implies a total pool of 40 participants would need to be included in the study. G*Power (version 3.1.2) [55] was used for these calculations.

In reviewing the literature, we were not able to identify data on the reliability of measurement of contraction of the transversus abdominis muscle on MRI (Fig. 3). The current study should be considered a pilot study for the impact of the motor control intervention for contraction of the transversus abdominis muscle on MRI.

For the secondary outcome of muscle size, data from our previous work of average lumbar multifidus cross-sectional area showed a correlation of 0.948 between repeated measures with a mean (SD) of 559 (72) mm^2^ [56]. With a total sample size of 36 participants, an effect size of 0.89% or 4.97 mm^2^ between groups (group*time interaction) should be detectable.

### Randomisation

Offsite randomisation procedures will ensure random and concealed allocation of participants. A researcher at La Trobe University who will have no contact with volunteers will randomise participants to either motor control and manual therapy (MCMT) or general strength and conditioning (GSC). A randomisation schedule (utilising block randomisation with random block lengths and stratification for gender) will be prepared in advance using a web-based randomisation program. Concealed allocation in accordance with the randomisation schedule will then be implemented by the researcher upon receipt of the name, gender and date of birth of a consenting participant to be enrolled into the trial.

### Study interventions

#### Motor control and manual therapy (MCMT)

Participants randomised to the MCMT group will receive twelve 30-min one-on-one physiotherapy sessions, consistent with previous protocols in chronic low back pain care [57, 58]. In the first 3 months ten sessions (one to two sessions a week) will be delivered with two sessions in the final 3 months. Treatment will take place at Advance HealthCare in Boronia by qualified physiotherapists, and will include a motor control exercise program in conjunction with the application of manual therapy based on the Specific Treatment of Problems of the Spine (STOPS) protocol [58]. Graded functional exercises will be implemented, targeting the local stabilising muscles transversus abdominis, multifidus and the pelvic floor muscles [59]. Progression of exercises will be on a pain-contingent basis. The program aims to restore optimal motor control during daily activities [59, 60]. Along with motor control exercises, participants will be guided in modifying maladaptive kinematics with postural correction [60].

Manual therapy treatment will be included at the discretion of the treating physiotherapists. Treatment will be in accordance with key principles for spinal manual therapy [61] and will include posterior-anterior and transverse mobilisation using rotation and soft tissue techniques of the lumbar and pelvic region. Basic cognitive-behavioural education will be utilised as required to assist participants to overcome any distress that may account in part for their altered motor control (such as inability to relax the abdominal wall, concerns about the safety of exercising). Attendance at treatments will be recorded and reported at the 3-and 6-months follow-up. A home-based exercise program will also be arranged for participants to complete between sessions, which will include motor control, postural control and pelvic floor exercises taught at each session, before gradually introducing the motor control skills into everyday activities.

#### General strength and conditioning (GSC)

Participants will complete a 6-month GSC training program, including gym-based supervised sessions and independent home-based exercises. An Accredited Exercise Physiologist (AEP) or Masters of Clinical Exercise Physiology students (supervised by an AEP) will administer the gym-based training in the Clinical Exercise Learning Centre (CELC), Deakin University, Burwood, Victoria or the Burwood YMCA at Deakin University. Participants will attend two 1-hour training sessions in weeks 1–12, and one to two training sessions per week in weeks 12–26. Sessions will consist of 20 min of aerobic conditioning, beginning at an intensity of 65–70% maximum heart rate (HR_max_) in the first 2 weeks and increasing to 65–85% HR_max_. This will be followed by progressive resistance training and proprioceptive exercises. Each session in the resistance program consists of five exercises from a selection of seven core exercises grouped according to an action: push, pull, trunk extension, trunk flexion and lift. The program follows training principles of undulating periodisation, varying loads, reps, sets and time under tension as shown in Table 1, Resistance training program overview. Progression will be determined with a time-contingent rather than pain-contingent manner, in line with previous studies on functional movement goals in perceived painful activity [62, 63]. Participants will receive pain education during the training sessions as required.

**Table 1:** Resistance training program overview

Week	Goal	Intensity	Frequency	Routine	Time under tension	Progression
1–4	Familiarisation, motor control and local muscular endurance	12–15 reps performed at 2 reps below volitional fatigue × 2 sets, 1 min rest between sets	2/wk	Full body	2 s concentric, 2 s eccentric	Once 2 sets of 15 reps at 2 consecutive training sessions are completed, resistance is increased
5–10	Muscular strength	6–10 reps performed at 2 reps below volitional fatigue × 2–3 sets, 2 min rest between sets	2/wk	Full body	2 s concentric, 2 s eccentric	Once 2 sets of 10 reps at 2 consecutive training sessions are completed, workload increases to 3 sets. Then progression made through increased resistance
11	Light week	10 reps at 80% of resistance used in the previous week × 3 sets, 2 min rest between sets	2/wk	Full body	2 s concentric, 2 s eccentric	None
12–15	Local muscular endurance	12–15 reps performed at 2 reps below volitional fatigue × 3 sets, 1 min rest between sets	1–2/wk	Full body	5 s concentric, 5 s eccentric	Once 3 sets of 15 reps at 2 consecutive training sessions are completed, resistance is increased
16–19	Muscular strength	6–10 reps performed at 2 reps below volitional fatigue × 3–4 sets, 2 min rest between sets	1–2/wk	Full body	2 s concentric, 2 s eccentric	Once 3 sets of 10 reps at 2 consecutive training sessions are completed, workload increases to 4 sets. Then progression made through increased resistance
20–25	Local muscular endurance	20–25 reps performed at 2 reps below volitional fatigue × 3 sets, 1 min rest between sets	1–2/wk	Full body	1–2 s concentric, 1–2 sec eccentric	Once 3 sets of 25 reps at 2 consecutive training sessions are completed, resistance is increased

*wk* week, *reps* repetitions, *s* seconds, *min* minutes

Attendance will be recorded along with exercises completed, session Rating of Perceived Exertion (RPE) and duration. Missed sessions will be noted with a reason, and a make-up session will be organised in the same week if possible. Independent home-based training will consist of three 20–40 min aerobic training sessions at 65–85% HR_max_ up to three times a week, consistent with studies on aerobic intensities for reducing pain [64–66]. Exercise selection is dependent on participant preference; however, for ease and compliance purposes, walking or jogging is recommended, followed by stretches taught in training sessions. Participants will be required to self-record their home exercises, which will be reviewed at 3 and 6 months follow-up. Secondly, participants are required to spend 5–10 min each day for at least the first 6 weeks completing mental rehearsal exercises of activities or actions that each individual perceives as injury- or pain-provoking. Progression of mental rehearsal tasks will be monitored, and exercise tasks replicating such movements will be introduced into the resistance program. Stretching exercises will be provided as individually required following the aerobic training component, at the end of the independent training.

### Outcome measures

Outcome measures will be obtained from participants at baseline and at 3 and 6 months. Trained research staff will adhere to the standardised procedures for all data collection to ensure consistency in equipment used in each test and documentation and storage of data collected.

#### Primary outcome measures

##### MRI

The evaluation of MRI images is to determine if particular exercise protocols have a positive or negative influence on IVD changes, muscular and vertebral function, motor control function and individual and overall muscle size and quality. Participants will be required to have an MRI scan at Imaging@OlympicPark at AAMI Park, Olympic Boulevard, on a separate day after testing at Deakin University. Due to known diurnal variation in the spine [67], all MRI scans will be performed after midday. Prior to the scan, the participant will rest in a sitting position for 20 min. The participant will remove any metallic objects from the body and clothing. A gown will be provided if needed. Participants lay in a supine position on the scanning bed, with a cushion wedge underneath their knees, hands above their heads. A Phillips Ingenia 3.0 T scanner (Philips Healthcare, NSW, Australia) will be used for all scans.

A series of scans will be run over 40 min, which include:

1. Sagittal T2 spin-echo multiecho, lumbar spine and lower thoracic spine; and sagittal T2-weighted scan. This will be used to determine the average lumbar spine intervertebral disc T2 relaxation time on MRI (Fig. 2)

**Fig. 2:**
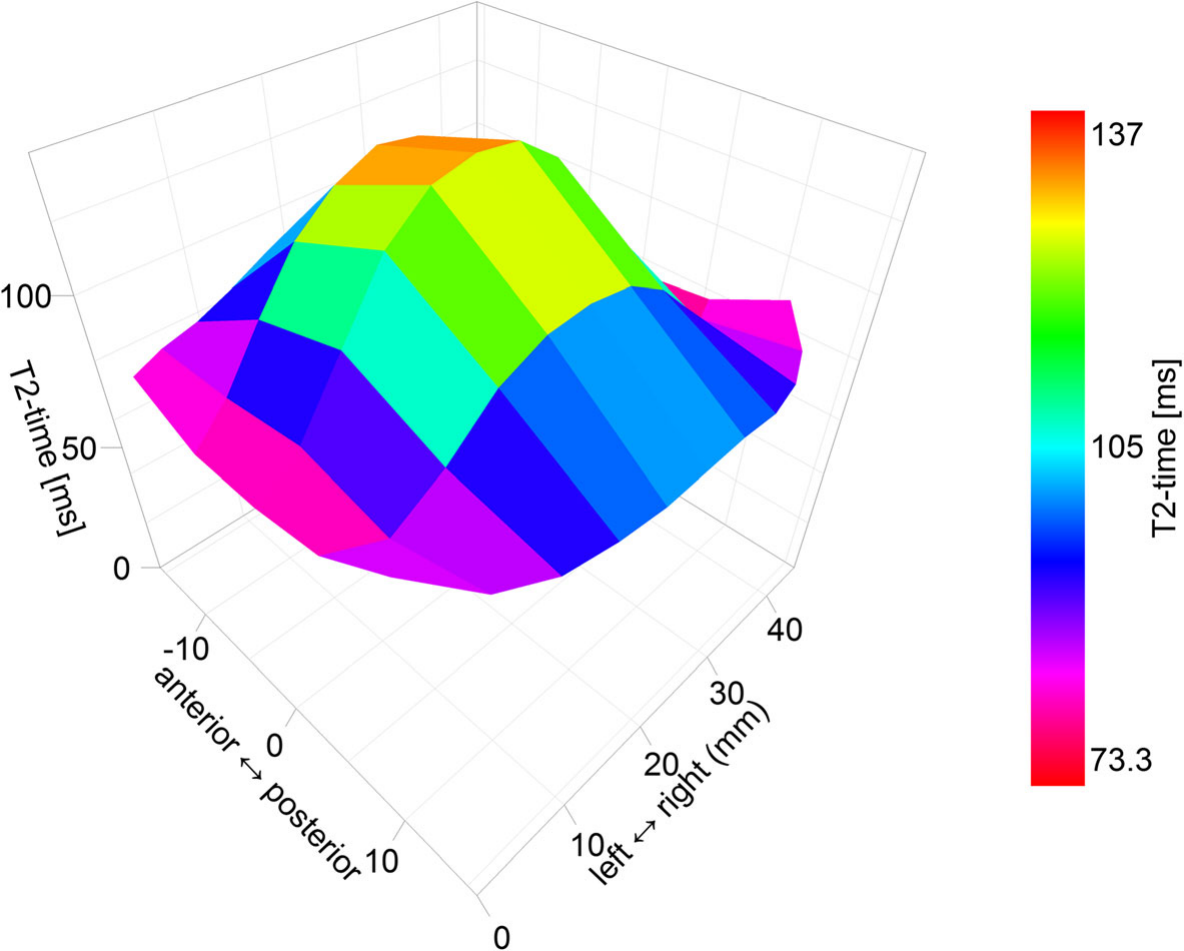
Intervertebral disc T2-time. Image shows the volumetric lumbar disc T2-time, averaged from all 5 lumbar discs [14]. Note that the T2-time is higher in the central nuclear portion of the disc, which is more hydrated. T2-time correlates with disc proteoglycan and water content [89]
2. Scan of the abdominal wall, to measure the change in thickness of the transversus abdominis muscle during a leg lift (Fig. 3)

**Fig. 3:**
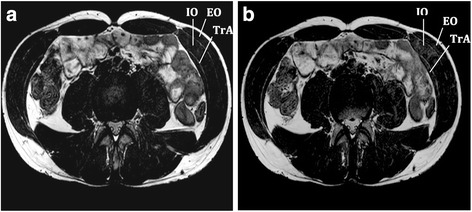
Contraction of the transversus abdominis muscle in a leg lift task. Image shows the transverse abdominis (*TrA*), internal obliques (*IO*) and external obliques (*EO*) at **a** rest and **b** contracted (leg lift)

The outcome assessors will be blinded to participant allocation and study time-point by assigning each MR data set a random code (obtained from www.random.org).

#### Secondary outcome measures

##### MRI

In addition to the scans for the primary outcomes, the following scans will also be included:

- Sagittal T2 spin-echo multiecho: for calculation of intervertebral disc volume and height
- Sagittal Dixon, lumbar and lower thoracic: for calculation of vertebral body fat content and vertebral body cortex form
- Axial Dixon, lumbar spine: for calculation of trunk muscle size and lumbar muscle fat content
- Sagittal diffusion weighted imaging (DWI): water diffusion rates in the intervertebral disc.

#### Physical function

##### Trunk extension and flexion endurance time

Muscular endurance of the trunk has been shown to reduce in patients with chronic low back pain [40], reducing the ability to maintain postures without pain and therefore decreasing physical activity levels. As the muscles of the trunk are directly involved in the movement of the lumbar region, measuring their endurance time can provide information on disability or functionality of the back. Local muscular endurance of the trunk will be assessed using a protocol that has been shown to be reliable, reproducible and safe for patients with chronic low back pain [68]. For trunk extension, participants will lie in a prone position with their umbilicus at the edge of the plinth bed, arms at their sides. They will be asked to lift their chest, legs and arms off the plinth, keeping hands away from the body, and tucking the chin in slightly. Maximal extension, where the chest remains completely off the plinth, is maintained until voluntary fatigue, where participants can no longer maintain their chest completely lifted, or participants are restricted by pain [68].

Flexion endurance will be measured in a supine position, with a 90° flexion at hips and knees. With arms across the chest, participants will be instructed to raise their head and shoulders up until the lowest part of the shoulder blades no longer rests on the plinth. Maximal flexion is held until voluntary fatigue, where shoulders can no longer be kept completely off the plinth, or pain limits performance [68]. Feedback on body position will be provided at 30-s intervals with instruction to adjust body position if necessary. Performance will be documented as time held to the nearest 0.01 s [68] using a portable stopwatch (Hart Sports, Aspley, Australia).

##### VO_2_ max

Decreased cardiovascular fitness is evident in low back pain [39]; therefore, measuring aerobic fitness can provide information as to whether being more aerobically fit has an additional benefit on treatment and management of chronic low back pain. A VO_2_ max test is a highly regarded measuring tool to determine aerobic capacity. Peak aerobic power will be assessed by a submaximal VO_2peak_ graded treadmill test (h/p/cosmos Quasar DE83365, Nussdorf‐Traunstein, Germany) using an individualised graded exercise test protocol, suitable for those with low exercise tolerance due to pain [69]. Participants warm up at a comfortable walking speed at 0% gradient, and speed is then gradually increased until a subjective Rating of Perceived Exertion (6–20 Borg point scale; RPE) [70] of 8/20 is achieved. The speed will remain constant for the duration of the exercise test. During the test the gradient is increased by 2% at the end of every minute until the participant subjectively reports a 17 (very hard) on the Borg RPE scale. The test is terminated at this point to avoid adverse signs and symptoms rather than reaching maximum volitional fatigue, which may exacerbate pain or increase withdrawal. This method of using RPE during graded exercise testing in chronic pain patients is reliable [71]. A 5-min active recovery will follow at 0% gradient. Expired respiratory gases will be collected through a breath-by-breath pneumotachography system (Innocor version 6.15, Innovision, Glamsbjerg, Denmark) that will be calibrated prior to each test as per equipment manufacturer guidelines.

The final 15-s average breath-by-breath interval for VO_2_, VCO_2_ and ventilation (VE) at each minute will be used for analysis. Heart rate (HR) will be measured and recorded at rest, throughout the test and during recovery at the end of every minute using an ear lobe pulse oximeter (Compatible 3 m, Nonin, MN, USA) [72]. Pre-exercise and post-exercise blood pressure will be measured using a manual sphygmomanometer and stethoscope. Submaximal exercise data will be used to predict maximal aerobic power using an individualised linear regression [73].

##### Muscular strength: one-repetition maximum (1RM) leg press

As with muscular endurance, overall muscular strength has been shown to decline with chronic low back pain [40]. Strength is an important component of muscular function, allowing everyday movements to be performed correctly without compensating muscle and causing injury. As the major muscles of the lower limb contribute greatly to trunk movement, as well as overall movement for everyday activities, a muscular strength test will be completed on the lower limbs using a 1RM 90° seated leg press (Synergy Omni leg press S‐31‐OPD, Yatala, Australia) [74, 75]. The participants will be instructed on correct technique and feet and seat position recorded for follow up testing. Prior to each test participants will complete ten repetitions for warm up at low load. The test will progressively increase in load by a minimum of 10 kilograms, with a rest period of 60-90 seconds between attempts, or until fully recovered. The 1RM is to be achieved within 8-10 attempts. If maximum weight (240 kg) is reached, participants will perform maximum repetitions up to10 repetitions. A predicted 1RM value will then be calculated [76]. The machine settings in relation to anatomy will be recorded and replicated in follow up tests.

##### Leg press muscular endurance

Endurance of the lower limb is also very important in the analysis of trunk function and disability, as it allows for maintenance for activities with less stress and pain. A muscular endurance leg press test of the lower limb will be performed using 70% of the achieved 1RM weight following a 2-min recovery period from completion of the muscular strength test. Maintaining machine setup and anatomical position, participants will perform their maximum number of repetitions at a tempo of 2 s eccentric and 2 s concentric pace, until volitional fatigue determined by resting at any point, or inability to complete a full repetition [75]. The baseline calculated weight will be replicated at the 3- and 6-month follow-up tests.

#### Dual energy X-ray absorptiometry (DXA)

##### Total body composition

Total body composition and bone mineral density will be assessed using an iDXA scanner (GE Lunar iDXA, Madison, WI, USA). Standard manufacturer procedures for quality assurance and quality control will be performed every measurement day. All participants will be required to remove all clothing, including bras, and jewellery and wear a surgical gown and underwear. Participants will be positioned centrally on the bed in supine position with palms facing down and ankles lightly strapped together, to ensure all limbs remain within the scan range. A full body scan will be used to assess body composition. The total body lean mass, fat mass and fat percentage will be reported as outcomes.

##### AP lumbar spine

Immediately after the total body scan, a second scan will be taken of the lumbar spine to determine lumbar bone mineral density. This is included to examine if particular exercise protocols influence bone density, and therefore if lumbar bone mineral density is a determinant of low back pain. For this, participants will remain in a supine position with a large square box placed underneath their legs to support a 90° position of the hip and knee, ensuring a flattened lumbar spine. The C-arm of the iDXA will be centred at L5 (+/–5 cm inferior to the umbilicus) and scan to T12. Scans will be repeated if the spine is not centred and straight, or the correct markers (iliac crest, vertebral body of T12 and ribs) are not visible in the scanning image. Lumbar spine areal bone mineral density will be reported as the outcome.

#### Transcranial magnetic stimulation (TMS)

TMS is used to measure excitatory and inhibitory responses in the connections between cortex and muscle and has been shown to be impacted in back pain [77]. Therefore, the inclusion of TMS in the outcomes is to determine if chronic pain influences nervous responses in the muscles of the back, and whether particular exercise protocols influence this function. Surface electromyographic (sEMG) activity of the left multifidus muscle will be recorded using bipolar disposable electrodes at the level of the L5 spinous process along the line joining the posterior superior iliac spine (PSIS) and the L1–L2 vertebral interspace according to the Surface ElectroMyoGraphy for the Non-Invasive Assessment of Muscles (SENIAM) guidelines [78], while the grounding electrode is positioned on the left iliac crest. All sEMG signals will be acquired, bandpass-filtered (amplification 1000×, bandpass filter 13–1000 Hz) and analysed using Powerlab 4/35 (AD Instruments, Bella Vista, Australia).

During the TMS procedure, all participants will be seated on a chair with their feet flat on the ground, with a slight forward lean to put the lumbar spine in lordosis. This method was used in previous TMS studies to induce a low-level tonic activation of the multifidus muscle, which eases the recording of motor evoked potentials (MEPs) from the multifidus muscles with minimal pain or discomfort [79, 80]. The mean rectified sEMG activity for the multifidus associated with this posture will be calculated, and an upper and lower target line will be set as guidelines to achieve a comparable level of muscle activation across all testing sessions. Any TMS trial in which the sEMG activity exceeds the target line will be rejected. A rest period of (≈120 s) will be provided, if necessary, between every 20 TMS stimuli to avoid fatigue or pain.

Single- and paired-pulse TMS will be applied using a double-cone coil (7 cm diameter per wing) connected to a BiStim 200^2^ magnetic stimulator (Magstim Co Ltd, Whitland, UK) over the optimal location on the primary motor cortex (M1) of the trunk muscles. The optimal scalp position for activation of the multifidus muscle will be determined from initial exploration over a 1-cm grid marked on a rubber cap worn over the participant’s head. The optimal scalp position is defined as the location that elicited the largest and most consistent MEP amplitude in at least 5 out of 10 TMS stimuli [81]. Once the optimal scalp location is found, single-pulse TMS will be used to determine the active motor threshold (AMT), which is defined as ≥200 μV in at least 5 out of 10 trials [81]. A TMS stimuli intensity that is 120% of AMT (1.2 AMT) will be used to index any change in corticospinal excitability.

Paired-pulse TMS using a conditioning: test stimulus paradigm will be used to measure changes in short-interval intracortical inhibition (SICI) [82]. This will be done by delivering a subthreshold conditioning stimulus (0.7 AMT) before a test TMS stimulus (1.2 AMT) that is separated by a 2-ms inter-pulse interval.

The parameters used for paired-pulse TMS to elicit SICI was adapted from a previous study [83]. To measure SICI, 10 unconditioned single-pulse test MEPs and 10 conditioned MEPs will be recorded in a randomised order and expressed as a ratio (SICI_ratio_) of the conditioned to the unconditioned single-pulse test MEP amplitude. Therefore, a lower SICI_ratio_ indicates a greater cortical inhibition.

Single-pulsed TMS will also be used to develop cortical maps of the back muscle. The cortical mapping technique will be performed by stimulating on the adjacent areas surrounding the hotspot over a 1-cm square grid [84]. Any changes in the area of the cortical maps will provide further indication of changes in brain excitability associated with the intervention, where shifts in the maps would indicate cortical reorganisation.

#### Anthropometry

##### Body mass index (BMI)

Height will be determined using a standard fixed stadiometer (Holtain Limited, Crymych, Pembs, UK) measured to the nearest 0.01 m with participants standing in anatomical position. Weight will be measured using calibrated scales (SECA 708, Hamburg, Germany) to the nearest 0.1 kg. Both measurements will be taken on the patient wearing a surgical gown with no footwear and all clothing removed except for underwear. A trained researcher will perform and assess the required measurements. BMI will be calculated to the nearest 0.1 kg/m^2^ using the standard formula: *body mass* (*kg*)/*height* ^*2*^ (*m* ^*2*^).

##### Waist circumference

Waist circumference will be measured by a trained researcher using a steel tape with the participant in a standing position. The tape will be placed horizontally around the participant’s waist immediately above the iliac crest according to the National Health and Nutrition Examination Survey (NHANES) III procedure [85]. Three measurements will be taken, and the mean of the two closest measurements will be used.

##### Hip circumference

Hip circumference will be measured using a steel tape measure with the participant in the standing position by a trained researcher. The tape will be placed horizontally around the widest part of the hips [86]. Three measurements will be taken, and the mean of the two closest measurements used.

### Self-reported measures

Self-reported measures of pain and disability will be included to determine if certain exercise protocols have a greater influence on an individual’s perception of treatment and the connection it has to physical changes. Online questionnaires included in the study will be the Subjective Complaints Questionnaire to determine the history of a participant’s low back pain, a visual analogue scale (VAS) for pain to monitor low back pain symptoms, the Sciatica Frequency and Bothersomeness Index to determine additional symptoms, the Centre for Epidemiologic Studies Short Depression Scale (CES-D 10) questionnaire for depressive symptomology, the Positive and Negative Affect Schedule (PANAS) to measure mood state, the Work Productivity and Activity Impairment questionnaire, the Sports Injury Rehabilitation Beliefs Survey (SIRBS) to examine factors that might influence treatment compliance, the International Physical Activity Questionnaire (IPAQ) to determine level of physical acitivity, a medication usage questionnaire, the Short Form-36 Health Survey (SF-36) V1 questionnaire on patient health and quality of life, the Oswestry Low Back Disability questionnaire, the Pittsburgh Sleep Quality Index Questionnaire, the Tampa Kinesiophobia Scale for fear of movement, the Endicott Work Productivity Scale, the Global Rating of Change Scale and a treatment satisfaction questionnaire to determine participants’ overall perception of and satisfaction with received treatment. Table 2 provides an overview of the questionnaires and delivery frequency.

**Table 2:** Study questionnaires and surveys

Document	Paper, baseline only	Paper, 0, 3, 6 months	Online, fortnightly	Online, 0, 3, 6 months	Completed by therapists at each session	Purpose
Subjective Complaints Questionnaire	X					History of the participant’s low back pain [90]
Work Productivity and Activity Impairment		X				Monitor work productivity and activity impairment [91]
VAS Pain Questionnaire			X			Monitor pain symptoms [92]
CES-D 10			X			Monitor depressive symptoms [93]
PANAS			X			Monitor mood state [94]
Global Rating of Change Scale				X		Participant’s overall perception of change since study commencement on a 7-point scale [95]
Sciatica Frequency and Bothersomeness Index				X		Measures the frequency and bothersomeness of a range of leg symptoms including pain, numbness, tingling and weakness [96]
Treatment satisfaction				X		Participant overall satisfaction with treatment, with results of treatment and with the prospect of enduring current symptoms for life [97]
SIRBS				X		Information on factors that might influence treatment compliance [98]
IPAQ				X		Physical activity questionnaire [99, 100]
Medication usage				X		Medication usage
SF-36 V1				X		Survey of patient health and quality of life [101]
Revised Oswestry Low Back Disability Questionnaire				X		Monitor disability related to back pain [102]
Pittsburgh Sleep Quality Index				X		Sleep quality [103]
Tampa Kinesiophobia Scale				X		Monitor fear of movement [104]
Endicott Work Productivity Scale				X		Monitor work productivity [105]
SIRAS					X	Log information about adherence to training [106]

*VAS* Visual analogue scale, *CES-D* Centre for Epidemiologic Studies Short Depression Scale, *PANAS* Positive and Negative Affect Schedule, *SIRBS* Sports Injury Rehabilitation Beliefs Survey, *IPAQ* International Physical Activity Questionnaire, *SIRAS* Sport Injury Rehabilitation Adherence Scale, *SF-36 V1* Short Form-36 Health Survey

### Data management and adverse event reporting

After initial telephone screening, all eligible participants will be given a unique code to be used for all forms and data collection to de-identify participants. Consent forms and allocation of identification numbers will be stored in a password-protected format on a secure Deakin University server. Coded data collection forms will be stored securely at Deakin University. For the duration of the study, data collected will be available only to participating researchers.

After the completion of the trial, dissemination of research results will only consist of aggregated data; individual participants will not be identified. Findings from this project will be released to relevant agencies and health care professionals that could potentially benefit from these findings. The study results will also be presented at national and international conferences and will appear in our annual reports and newsletters. Should participants wish to have access to their corresponding results, a brief report via email or post will be provided and copies of published articles will be sent via email or post at the end of the study. Information on paper copy, computer or CD will be stored for 15 years from the date of publication. After a period of 15 years from the date of any publication of the results from the study, paper copies of individual responses will be disposed of.

If an adverse event occurs, then the adverse event reporting form will be completed. The participant will be advised to contact his/her medical practitioner where necessary.

### Statistical analysis

Mixed effects models with an intention-to-treat approach will be performed for all continuous variables to assess the interaction effect of the intervention over time (group × time). All statistical analyses will adopt a significance level of 0.05.

## Discussion

It is well accepted that exercise is effective for reducing pain in those suffering chronic low back pain. Furthermore, reviews of various exercise approaches have revealed little differences in the impact on reducing pain between common exercise interventions including specific spinal exercises or general conditioning [87, 88]. However, the literature on this subject tends to solely focus on the effects of exercise on subjective pain levels. Additional deficits evident in low back pain have little data on the effects that certain exercise protocols have, which may contribute to successful treatment and management of chronic low back pain. This study aims to investigate and compare two treatment approaches for chronic low back pain that represent two distinct loading strategies. This trial will be one of the first to extensively examine the impact of different treatment approaches on multiple outcome domains and side effects in people with chronic low back pain. Although some outcome measurements used in this study are not easily replicable in clinical practice, the results of this study will help us understand the effect that different exercises have on various contributors to chronic low back pain, not just the pain itself. They will also help us determine if particular exercises are more favourable over general activity and movement. This study promises to support clinical practice by providing outcome-based evidence on the effectiveness of different exercises, and to help guide treatment decisions and exercise plans for patients with chronic low back pain.

### Trial status

At the time of submission, participant recruitment is in process, but is not yet complete.

## Additional file

Additional file 1: SPIRIT 2013 checklist: recommended items to address in a clinical trial protocol and related documents. (DOC 121 kb)

## Abbreviations

1RM: One-repetition maximum
BMI: Body mass index
DXA: Dual energy X-ray absorptiometry
GSC: General strength and conditioning
IVD: Intervertebral disc
LME: Local muscular endurance
MCMT: motor control and manual therapy
MRI: Magnetic resonance imaging
TMS: Transcranial magnetic stimulation
